# Comparing the effect of sedation with dexmedetomidine and propofol on sleep quality of patients after cardiac surgery: A randomized clinical trial

**DOI:** 10.34172/jcvtr.33086

**Published:** 2024-09-20

**Authors:** Rasoul Azarfarin, Mohsen Ziaei Fard, Maryam Ghadimi, Yasmin Chaibakhsh, Marziyeh Yousefi

**Affiliations:** Rajaie Cardiovascular Medical and Research Center, Iran University of Medical Sciences, Tehran, Iran

**Keywords:** Dexmedetomidine, Propofol, Sleep quality, Cardiac surgery

## Abstract

**Introduction:**

Sleep quality is the main concern of patients after cardiac surgery. We compared the effect of two routinely used sedatives on the sleep quality of patients admitted to the intensive care unit (ICU) after cardiovascular surgery.

**Methods:**

It is a prospective, controlled, randomized clinical trial. A total of 120 patients, after cardiac surgery were enrolled. During extubating, patients were randomized into two groups: 60 patients received an infusion of dexmedetomidine (precede; 0.5 μg/kg/h), and 60 patients received 50 μg/kg/min propofol for 6 hours. Baseline characteristics were compared between the groups. The patients completed the St. Mary’s Hospital Sleep Questionnaire, and the scores were compared between the groups.

**Results:**

The groups were not different in terms of demographics, underlying diseases, smoking/drug abuse/alcohol, number of vessels involved, history of non-cardiac surgery, and mean levels of serum parameters (*P*>0.05). Most of the medications used were similar between the groups (*P*>0.05), except calcium channel blockers (more frequently used in the propofol group [*P*=0.027). The details of surgery were not statistically significant different (*P*>0.05); but, the mean volume of platelet received after the surgery was higher in propofol group (*P*=0.03). The propofol group had less problems with last night’s sleep (0 vs 0.1±0.66), felt more clear-headed (4.9±0.6 vs 4.68±0.58, were more satisfied with their last night’s sleep (52.1% vs 47.9%), but spent more time getting into sleep (0.38±1.67 vs 0 ) (*P*<0.5).

**Conclusion:**

The sleep quality of patients under the influence of propofol seemed to be better than dexmedetomidine after cardiac surgery.

## Introduction

 Cardiovascular diseases (CVDs) are the leading cause of mortality worldwide, and cardiac surgery is frequently performed around the world. Like other types of surgery, cardiac surgery has multiple postoperative complications.^1^ One of the complications after cardiac surgery is poor sleep during hospitalization, especially in the intensive care unit (ICU), which may persist for several months after discharge.^2^ Factors related to sleep disturbances include physical factors (including pain, dyspnea, nocturia, and poor cardiac function), environmental factors (including noise, light, mechanical ventilation, and other procedures), psychological factors (including anxiety and depression), and individual factors, such as age and sex.^3^

 Sleep is a periodic and reversible disengagement from the environment, which is essential for rest and repair. It has an important role in cardiovascular function, and sleep disturbances can cause or worsen anxiety, irritability, and anger, change the cardiac rhythm and myocardial oxygen demand, and impair recovery from disease.^4^ Sleep deprivation may also result in delirium in the patients.^5^ Several therapeutic methods have been suggested for improving the sleep quality of patients after cardiac surgery; non-pharmacological interventions include the use of earplugs and eye masks with relaxing background music,^6^ acupuncture;^7^ also, medications, including melatonin and benzodiazepines (like midazolam or lorazepam), have been suggested.^8^ However, pharmacological medications have their own complications and contra-indications and cannot be prescribed for all patients, and the efficacy of non-pharmacological interventions has not been confirmed yet. Therefore, research is continued on the methods that can improve sleep quality after cardiac surgery.

 Current guidelines suggest the priority of sedation strategies using non-benzodiazepine sedatives over sedation with benzodiazepines for mechanically-ventilated patients in the ICU; the two most common sedatives currently used include propofol or dexmedetomidine (DEX).^5,9^ Propofol is an intravenous (IV) lipid-soluble medication with sedative, hypnotic, anxiolytic, amnestic, antiemetic, and anticonvulsant properties. It crosses the blood-brain barrier and binds to several receptors of the central nervous system with a short duration of effect.^10^ Its advantages over benzodiazepines include lack of accumulation, quick onset, easy adjustment, and fast recovery after discontinuation.^11^ However, the adverse effects of propofol limit its use (which include pain on injection, hypotension, bradycardia, respiratory depression, and hypertriglyceridemia). Propofol infusion syndrome is also rare; but life-threatening adverse effect that remains an important concern for propofol use.^12^ DEX is a novel sedative analgesic, selective α_2_ agonist, rapidly redistributed, with easily arousable action, minimal respiratory depression, a slight decrease in blood pressure, and a modest reduction in heart rate.^13^ It reduces the circulating catecholamines, and its opioid-sparing effect may also reduce opioid requirements in critically ill patients.^14^

 Studies have compared different aspects of DEX with propofol, administered in the ICU, for post-cardiac sedation, which have shown the priority of DEX in reducing postoperative delirium and length of intubation compared with propofol; although adverse effects such as the higher risk of bradycardia have also been reported for DEX.^15^ Intraoperative awareness and recall,^16^ length of mechanical ventilation, pain, and opioid use, hemodynamics, and acute kidney injury are the other aspects compared in the recent literature between propofol and DEX.^17^ However, it is not known whether DEX is better than propofol for sleep quality in postcardiac patients admitted to ICU. Therefore, in this prospective, randomized study, we compared the effect of DEX and propofol on the sleep quality of patients admitted to the ICU after cardiovascular surgery.

## Materials and Methods

 This study was conducted on 120 patients admitted to the ICU after cardiac surgery at Rajaei Cardiovascular Medical and Research Center, Tehran, Iran. Informed written consent was obtained from all of the patients. The study protocol was registered in the Iranian Registry of Clinical Trials “https://irct.ir” under the code “IRCT20161127031131N3” and approved by the Iran Institutional Ethic Committee under the code “IR.RHC.1400.048”.

 Patients after cardiovascular surgery aged above 18 years were included in the study. The sample size of this study was calculated at 59 in each group, based on the study by Yang and colleagues,^18^ considering the frequency of poor sleep after cardiac surgery at 82.8% and assuming 205 reduction by DEX; online sample size calculator was used: https://select-statistics.co.uk/calculators/sample-size-calculator-two-proportions/ and considering confidence level at 90%, study power at 80%, and error type 1 at 5%. Accordingly, we recruited 60 patients into each group. The patients were not included in the study if they had a prior solid organ transplant, were pregnant or lactating, had an acute severe neurological disorder, had atrioventricular-conduction block grade II or III (unless a pacemaker installed), patients using alpha-2 agonists or antagonists within 24 hours before the operation. Patient with a history of alcohol consumption or administration of medicine for sleep problem such as melatonin and benzodiazepines were also excluded.

 All patients were given general anesthesia, induced by midazolam (5-10 mg/kg), fentanyl (250-500 μg/kg) or sufentanil (25-50 μg/kg), and rocuronium bromide (0.6-1.2 mg/kg). An arterial line (20-gauge plastic cannula) was inserted for invasive blood pressure monitoring in the left radial artery or in the non-dominant hand. For maintenance, midazolam (1 μg/kg/min), atracurium (10 μg/kg/min), and fentanyl (0.1 μg/kg/min) doses were infused for 3 to 4 hours. They underwent cardiac surgery (coronary or valvular). At the end of the operation, the patients were admitted directly to the cardiothoracic ICU, mechanically ventilated, and assessed for 12 hours in the postoperative period. The patients were ventilated by the volume-assist control mode with a tidal volume of 7-8 mL/kg of predicted body weight. The fraction of inspired oxygen (FIO_2_) and respiratory rate adjustments were made according to routine blood-gas analyses to maintain the partial pressure of arterial oxygen at 80-100 mmHg and partial pressure of arterial carbon at 35-40 mmHg.

 Patients were randomly divided into two groups; randomization was done in the form of quadruple blocks using the randomization table extracted from www.randomization.org, and patients were randomly assigned to each of the two study groups. The starting maintenance infusion fentanyl doses were 50 μg//kg/h in patients in either group after being admitted to the cardiothoracic ICU. At the time of extubation, one group received an infusion dose of DEX (loading dose of 1µgr/Kg IV Infusion over 10 minutes and followed by 0.5 μg/kg/h IV infusion), and the other group received 50 μg/kg/min propofol for 6 hours. The patients were excluded if they received both DEX and propofol concomitantly for the primary sedation or an alternative agent as the primary sedation, had mean arterial pressure (MAP) < 55 mm Hg (despite appropriate intravenous volume replacement and vasopressors), HR < 50/min, and/or atrioventricular-conduction block Grade II or III (despite pacemaker installed). Of note all drugs mentioned in the current study were purchased from Aburaihan inc. Tehran. Iran

 Sleep quality was evaluated using the Saint Mary’s Hospital Sleep Questionnaire (SMHSQ), designed in 1981 by Ellis and colleagues.^19^ This questionnaire has 14 questions: questions 1-4, questions 7, 8, and 14 ask about the duration of different sleep parameters (hours and minutes). Other questions are multiple-choice and scored; higher scores indicate better sleep quality. This questionnaire evaluated different aspects of sleep, including sleep depth, sleep latency, sleep quality, and awakening. The psychometric validation of this questionnaire has been evaluated and approved previously.^20,21^ The patients were asked to complete this questionnaire 1 day after the extubation while they were resting in their bed. The patients who had completed the questionnaire were asked not to contact the other patients who had not so that they would not share their ideas in this regard.

###  Statistical analysis

 The collected data were organized, tabulated, and statistically analyzed using the statistical software IBM SPSS Statistics for Windows version 22.0 (IBM Corp. 2013. Armonk, NY: IBM Corp.) The qualitative data were reported by frequency (percentage) and compared between two groups using the Chi-squared test. For the numeric variables, first, the normal distribution of data was evaluated using One-sample Kolmogorov-Smirnov test; after confirmation of normal distribution, they were reported using mean and standard deviation and compared between the groups using independent samples t-test. Statistical significance was *P* < 0.05 for the interpretation of the results of tests of significance.

## Results

 Of the total of 120 patients, 60 were evaluated in the DEX group and 60 in the propofol group. The demographics, underlying diseases, and smoking/drug abuse/alcohol were not different between the two study groups (*P* > 0.05; Table 1). Of all patients included in this study, none had chronic obstructive pulmonary disease (COPD). Furthermore, the clinical characteristics, including the number of coronary vessels involved and history of non-cardiac surgery, were not different between the two study groups (*P* > 0.05; Table 1). The medical history of patients also showed that most medications were not different between the groups (*P* > 0.05; Table 2), except calcium channel blockers, which were more frequently used in the propofol group (*P* = 0.027).

**Table 1:** Comparison of the baseline characteristics between the two study groups

	**Variable **	**Categories **	**Total**	**Dexmedetomidine Group** **n=60**	**Propofol Group** **n=60**	***P*** ** value**
Demographics	Sex, n (%)	Male	73	35 (47.9)	38 (52.1)	0.739*
		Female	47	24 (51.1)	23 (48.9)	
	Age (years), mean ± SD		-	55.27 ± 15.79	54.62 ± 14.35	0.813^†^
	Weight (kg), mean ± SD		-	74.92 ± 13.27	73.66 ± 13.97	0.614^†^
	Height (cm), mean ± SD		-	164.81 ± 9.15	165.85 ± 9.12	0.535^†^
Underlying diseases	Diabetes mellitus, n (%)	No	81	39 (48.1)	42 (51.9)	0.748*
		Yes	39	20 (51.3)	61 (50.8)	
	Hypertension, n (%)	No	42	23 (54.8)	19 (45.2)	0.368*
		Yes	78	36 (46.2)	42 (53.8)	
	Atrial fibrillation, n (%)	No	115	56 (48.7)	59 (51.3)	0.621*
		Yes	5	3 (60)	2 (40)	
	Dyslipidemia, n (%)	No	84	38 (45.2)	46 (54.8)	0.189*
		Yes	36	21 (58.3)	15 (41.7)	
	Renal failure, n (%)	No	118	57 (48.3)	61 (51.7)	0.147*
		Yes	2	2 (100)	0	
	Cerebrovascular accident, n (%)	No	117	58 (49.6)	59 (50.4)	0.579*
		Yes	3	1 (33.3)	2 (66.7)	
Smoking/ Alcohol/ drug abuse	Smoking, n (%)	No	102	52 (51)	50 (49)	0.344*
		Yes	18	7 (38.9)	11 (61.1)	
	Alcohol, n (%)	No	119	59 (49.6)	60 (50.4)	0.323*
		Yes	1	0	1	
	Opium addiction, n (%)	No	107	53 (49.5)	54 (50.5)	0.818*
		Yes	13	6 (46.2)	7 (53.8)	
Clinical	Number of coronary vessels involved	0	87	43 (49.4)	44 (50.6)	0.801*
		2	3	2 (66.7)	1 (33.3)	
		3	30	14 (46.7)	16 (53.3)	
	History of non-cardiac surgery	No	97	48 (49.5)	49 (50.5)	0.886*
		Yes	23	11 (47.8)	12 (52.2)	

*The results of Chi-square test, ^†^The result of independent samples t-test

**Table 2:** Comparing the frequency of the cardiac medications used between the two study groups

**Variable **	**Categories **	**Total**	**Dex Group** **n=60**	**Propofol group** **n=60**	***P*** value*
Statin, n (%)	No	45	22 (48.9)	23 (51.1)	0.962
	Yes	75	37 (49.3)	38 (50.7)	
Beta blocker, n (%)	No	51	27 (52.9)	24 (47.1)	0.477
	Yes	69	32 (46.4)	37 (53.6)	
ACE inhibitor, n (%)	No	55	23 (41.8)	32 (58.2)	0.139
	Yes	65	36 (55.4)	29 (44.6)	
Calcium channel blockers, n (%)	No	106	56 (52.8)	50 (47.2)	0.027
	Yes	14	3 (21.4)	11 (78.6)	
Aspirin, n (%)	No	40	20 (50)	20 (50)	0.897
	Yes	80	39 (48.8)	41 (51.3)	
Heparin, n (%)	No	114	55 (48.2)	59 (51.8)	0.379
	Yes	6	4 (66.7)	2 (33.3)	
Nitrate, n (%)	No	88	43 (48.9)	45 (51.1)	0.912
	Yes	32	16 (50)	16 (50)	
Diuretic, n (%)	No	101	47 (46.5)	54 (53.5)	0.184
	Yes	19	12 (63.2)	7 (36.8)	
Plavix, n (%)	No	81	43 (53.1)	38 (46.9)	0.216
	Yes	39	16 (41)	23 (59)	

*The results of Chi-square test

 Comparing the mean serum levels of the laboratory parameters showed no difference between the two study groups (*P* > 0.05; Table 3). The surgery-related factors are compared in Table 4, which shows there was no difference between the groups in terms of the type of surgery, aortic cross-clamp time, cardiopulmonary bypass time, packed red blood cell (RBC), epinephrine and norepinephrine infusion dosage, tracheal intubation time, mean volume of fresh frozen plasma (FFP) received during surgery or mean volume of FFP and platelet in ICU. However, the mean volume of platelets received after surgery was higher in group 2 (*P* = 0.03; Table 4).

**Table 3:** Comparing the results of laboratory tests between the two study groups

**Variable **	**Categories **	**Unit **	**Dexmedetomidine Group** **n=60**	**Propofol Group** **n=60**	***P*** value*
White blood cell		mg/dL	7027.81 ± 2252.5	7634.43 ± 20.99.5	0.130
Platelet count × 1000		mg/dL	218.12 ± 50.55	215.67 ± 56.52	0.803
Hemoglobin level, Number	≤ 12	mg/dL	16	21	0.429
	> 12		44	39	
Estimated sedimentation ratio, Number	≤ 30	mg/dL	13	15	0.829
	> 30		47	45	
Creatinine		mg/dL	1.11 ± 0.76	1.10 ± 0.35	0.965
Blood urea nitrogen		mg/dL	15.76 ± 5.08	16.93 ± 7.03	0.300
Serum glutamic-oxaloacetic transaminase (SGOT)		mg/dL	22.29 ± 9.32	21.74 ± 10.92	0.767
Serum glutamic-pyruvic transaminase (SGPT)		mg/dL	22.20 ± 12.81	20.85 ± 10.15	0.523
Alkaline phosphatase		mg/dL	171.07 ± 45.52	159.46 ± 45.34	0.164
Prothrombin time		mg/dL	18.95 ± 18.37	15.58 ± 3.60	0.162
Partial thromboplastin time		mg/dL	101.18 ± 450.55	37.41 ± 10.88	0.271
International normalized ratio		mg/dL	1.15 ± 0.27	1.15 ± 0.12	0.855

*The result of independent samples t-test; values are reported as mean ± standard deviation

**Table 4:** Comparing the surgery-related factors between the two study groups

**Variable **	**Categories **	**Total**	**Dexmedetomidine Group** **n=60**	**Propofol Group** **n=60**	***P*** value*
Type of surgery	Emergency	54	30 (55.6)	24 (44.4)	0.205*
	Elective	66	29 (43.9)	37 (56.1)	
Operation type	Coronary surgery	102	51 (50)	51 (50)	0.664*
	Valve surgery	18	8 (44.4)	10 (55.6)	
Aortic cross-clamp time (min), mean ± SD		-	66.08 ± 33.88	71.51 ± 32.21	0.371
Cardiopulmonary bypass time (min), mean ± SD		-	107.54 ± 48.38	112.07 ± 43.26	0.590
Packed red blood cell unit, mean ± SD		-	0.76 ± 0.99	0.56 ± 0.72	0.195
Fresh frozen plasma unit (cc)		-	0.71 ± 1.14	1.08 ± 1.02	0.064
Platelet unit (cc), mean ± SD		-	0.66 ± 1.12	1.10 ± 1.08	0.030
Epinephrine infusion dosage (µgr/Kg/min), mean ± SD		-	0.17 ± 0.38	0.17 ± 0.38	0.962
Norepinephrine infusion dosage (µgr/Kg/min), mean ± SD		-	0.03 ± 0.18	0	0.150
Tracheal intubation time, mean ± SD		-	11.44 ± 2.79	11.57 ± 2.84	0.796
ICU-packed cell unit, mean ± SD		-	0.07 ± 0.25	0.20 ± 0.44	0.053
ICU-fresh frozen plasma unit, mean ± SD		-	0.03 ± 0.260	0.03 ± 0.180	0.978
ICU-platelet unit, mean ± SD		-	0	0.05 ± 0.218	0.086

*The results of the Chi-square test, ^†^The result of independent samples t-test

**Figure 1:**
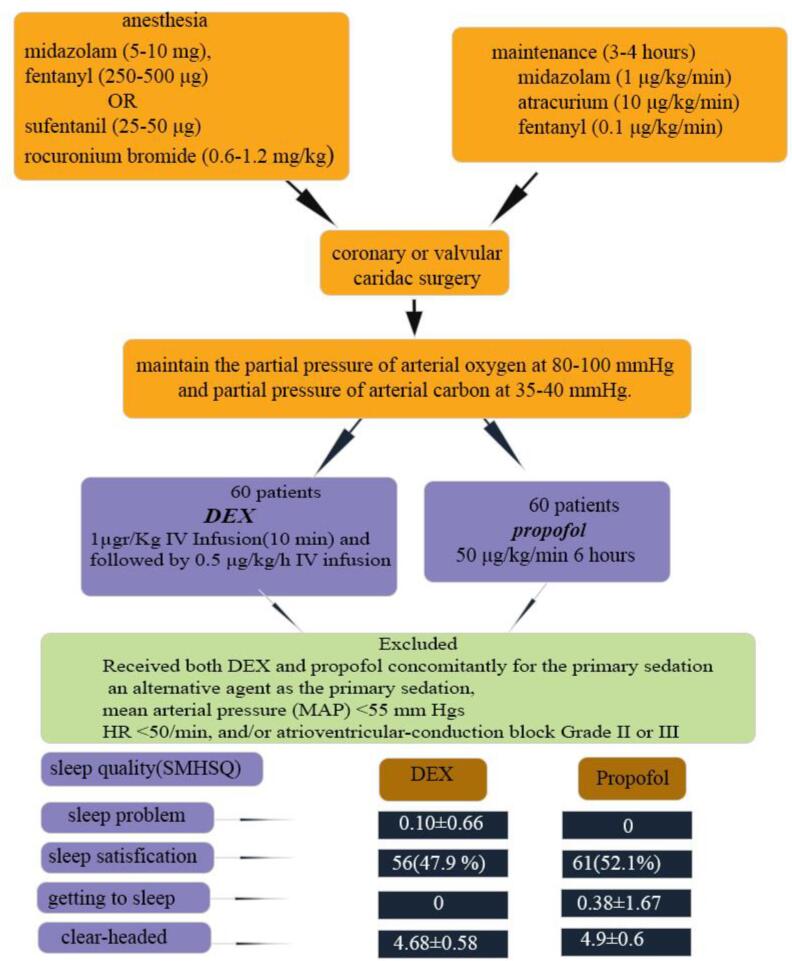
schematic representative of study

 Considering the questions of the sleep quality questionnaire, the propofol group less more problems in last night’s sleep, felt more clear-headed, were more satisfied with their last night’s sleep, but spent more time getting into sleep (*P* < 0.05; Table 5). Figure 1 indicated the graphic abstract of this study (Figure 1).

**Table 5:** Comparing the Q between the two study groups

**Variable**	**Dexmedetomidine Group** **n=60**	**Propofol group** **n=60**	***P*** value*
Q5. How was your sleep depth?	5.73 ± 1.42	6.02 ± 1.23	0.42
Q6. How many times did you wake up?	2.81 ± 1.04	2.16 ± 1.10	0.98
Q7. How much did you sleep last night?	6.76 ± 1.36	6.95 ± 1.23	0.41
Q8. How much did you sleep yesterday?	8.75 ± 1.55	8.97 ± 1.24	0.34
Q9. How well did you sleep last night?	4.39 ± 0.67	4.68 ± 0.78	0.79
Q9b. If not, what was the problem?	0.10 ± 0.66	00	**0.01**
Q10. How clear-headed did you feel after getting up this morning?	4.68 ± 0.57	4.90 ± 0.60	**0.04**
Q11. How satisfied were you with your last night’s sleep?	3.88 ± 0.65	4.11 ± 0.37	**0.008**
Q12. Were you troubled waking early and getting to sleep again?	YES: 56(47.9)	YES: 61(52.1)	0.074
	NO: 3(100)	NO: 0	
Q13. How much difficulty did you have in getting to sleep last night?	1.61 ± 0.49	1.39 ± 0.59	0.52
Q14. How long did it take you to fall asleep last night?	0.00	0.38 ± 1.67	**<0.001**

*The results of the Chi-square test, ^†^The result of independent samples t-test

## Discussion

 Comparing the two groups, sedated with propofol and DEX, showed that the groups were similar in terms of demographics, underlying diseases, medications used, smoking/drug abuse/alcohol, and laboratory tests, as well as the clinical characteristics and surgery-related factors. These results showed that the groups were comparable, and these factors did not confine with the main results. Evaluating the scores of the questionnaire showed that the groups had differences in several aspects of sleep quality. According to the results, the DEX group had fewer problems in last night’s sleep, felt less clear-headed, and spent less time getting to sleep, although they were less satisfied with their sleep compared with the propofol group. Therefore, based on the results of the present study, DEX does not seem to be significantly superior to propofol in terms of the sleep quality of postcardiac patients admitted to ICU. The mechanism of this effect has not been investigated in this study. However, previous studies have investigated the effect of these two medications on brain functions related to sleep.

 The sleep cycle is divided into non-rapid eye movement (NREM) and rapid eye movement (REM); NREM is divided into three stages: N1, N2 and N3. Evidence has suggested that moderate sedation with DEX produces changes (slow-delta oscillations and spindles) in the electroencephalogram (EEG), similar to that occurring during stage two of non-REM sleep; deeper sedation produces strong slow-delta activity without spindles, similar to stage three non-REM sleep.^22^ Therefore, the restorative brain state produced by DEX could improve the sleep quality of patients. Propofol also improves subcortical sleep pathways through γ-amino-butyric acid (GABA)-ergic inhibitory interneurons. Both propofol and DEX may modulate bottom-up pathways as well. Brain functional study suggests greater cortical effect for propofol, considering changes in brain connectivity; preservation of thalamic connectivity with key nodes of arousal and saliency detection network during N3 sleep may explain the rapid recovery of oriented responsiveness, observed under DEX sedation, while the default mode network is altered during propofol-induced unresponsiveness. The same mechanism (more natural restorative sleep-promoting action) has been suggested as the mechanism for reduced risk of delirium, compared with propofol.^23^ EEG evaluation of the patients, anesthetized with propofol, showed that the slow waves produced by propofol administration were similar to that observed during NREM sleep.^24^ These mechanisms may justify the difference in sleep quality of patients after cardiac surgery in the ICU. But further studies are required to demonstrate the exact mechanism of these two drugs on the sleep quality of this specific group, as the causes of sleep disturbance in the ICU are different from overall sleep in other conditions.

 Considering the insufficient evidence available on the effect of propofol and/or DEX on sleep quality in post-cardiac surgery patients in the ICU, we challenge our results with studies evaluating the effect of each of these two sedatives on sleep quality of patients admitted to the ICU. A review of studies on the effect of medications used for improving sleep in the ICU has also claimed that the effect of propofol on improving the quality and quantity of sleep remains debatable.^25^ These results are mainly because of the different tools used for the measurement of sleep; some have used polysomnography, others Ramsay sedation scale,^26^ Pittsburg Sleep Diary, or Bispectral Index;^27,28^ none used the tool used in the present study for evaluation of sleep quality. In one randomized study, it has been shown that propofol improved sleep quality and structure of sleep better than flunitrazepam, a benzodiazepine, in patients in ICU.^29^ The results of this study is consistent with the results of the present study, considering the favorable effect of propofol on sleep in the ICU, but the compared medication and assessment tool were different. Also, in our study, the patient groups consisted of post-cardiac surgery patients. Others reported no difference in total sleep time and sleep efficiency with disrupted REM sleep in the propofol group vs. no-propofol.^30^ But these results were obtained in mechanically-ventilated critically ill patients, who have different physical and psychological states, compared with our study group. Also, critically ill patients have limited communication to discuss their sleep quality.

 The effect of DEX on sleep quality in the ICU has also been evaluated, although not compared with propofol. A review of studies on this issue has shown different assessment tools used for sleep quality. One study that used SMHSQ showed that DEX could improve postoperative sleep quality, compared with saline, while patients felt more light-headed after getting up; no difference was observed in sleep satisfaction.^31^ The higher light-headedness documented in this study is consistent with the results of the present study, although the study population differed, as Mao and colleagues evaluated patients undergoing lateral thoracotomy for thoracic esophageal cancer, not admitted to ICU. Polysomnographic studies have also revealed improved sleep quality/efficiency in non-mechanically^32,33^ or mechanically ventilated critically ill patients.^34^ Another study has also reported contrary results by polysomnography evaluation of mechanically ventilated patients. They reported severely disturbed sleep architecture with no effect on slow wave or REM sleep by DEX.^35^ The results of this study seem more consistent with the present study. However, these studies have not used SMHSQ and have not compared their results with propofol to be comparable with the results of the present study. The patient group studied was also different from that of ours. In addition to the above, other factors, such as the time of surgery (day or night) may also influence the effect of DEX on the sleep quality of patients.^36^

 The main strength of the present study was the novelty in comparing these two medications, which are the commonest sedatives used in the ICU, while the lack of similar studies hindered the appropriate challenge of the results with similar studies. The similar baseline, clinical, and surgery-related factors of the two groups were another strength of the present study, which confirmed the accuracy of randomization and reduced the effect of confounders on the results of the study. However, this study had some limitations, as well. One major limitation is attributed to the nature of the outcome measure; sleep quality is a subjective matter, and any bias in the patients’ answers to the questions can influence the results of this study. Another limitation could be related to the selection of participants from one center, which increases the risk of the influence of confounders on the results.

## Conclusion

 Comparing the effect of two sedatives administered in the ICU to post-cardiac surgery patients showed that DEX was not significantly superior to propofol in some aspects, while propofol appeared superior to DEX in overall satisfaction from last night’s sleep. As this study was the only one to evaluate this issue, further studies are required to demonstrate the different aspects of sleep in the ICU in these specific groups of patients and the effect of these two medications on sleep quality.

## Acknowledgements

 The authors would like to thank the Rajaie Cardiovascular Medical and Research Center for its support and all the nurses working in the adult ICUs of Rajaei Hospital for their cooperation in data collection.

## Competing Interests

 No competing interest

## Ethical Approval

 The study protocol was registered in the Iranian Registry of Clinical Trials “https://irct.ir” under the code “IRCT20161127031131N3” and approved by the Iran Institutional Ethic Committee under the code “IR.RHC.1400.048”.
